# *In vivo* and *in vitro* infection dynamics of honey bee viruses

**DOI:** 10.1038/srep22265

**Published:** 2016-02-29

**Authors:** Jimena Carrillo-Tripp, Adam G. Dolezal, Michael J. Goblirsch, W. Allen Miller, Amy L. Toth, Bryony C. Bonning

**Affiliations:** 1 Department of Ecology, Evolution, and Organismal Biology, Iowa State University, Ames, IA 50011, USA; 2 Department of Plant Pathology and Microbiology, Iowa State University, Ames, IA 50011, USA; 3 Department of Entomology, Iowa State University, Ames, IA 50011, USA; 4 Department of Entomology, University of Minnesota, St. Paul, MN 55108, USA

## Abstract

The honey bee (*Apis mellifera*) is commonly infected by multiple viruses. We developed an experimental system for the study of such mixed viral infections in newly emerged honey bees and in the cell line AmE-711, derived from honey bee embryos. When inoculating a mixture of iflavirids [sacbrood bee virus (SBV), deformed wing virus (DWV)] and dicistrovirids [Israeli acute paralysis virus (IAPV), black queen cell virus (BQCV)] in both live bee and cell culture assays, IAPV replicated to higher levels than other viruses despite the fact that SBV was the major component of the inoculum mixture. When a different virus mix composed mainly of the dicistrovirid Kashmir bee virus (KBV) was tested in cell culture, the outcome was a rapid increase in KBV but not IAPV. We also sequenced the complete genome of an isolate of DWV that covertly infects the AmE-711 cell line, and found that this virus does not prevent IAPV and KBV from accumulating to high levels and causing cytopathic effects. These results indicate that different mechanisms of virus-host interaction affect virus dynamics, including complex virus-virus interactions, superinfections, specific virus saturation limits in cells and virus specialization for different cell types.

Honey bees share a long historical relationship with humans^1^ and are among the most economically important beneficial insects because of their pollination services^2^. Recent declines in wild and managed bees have led the scientific community to focus on possible causes for these declines and virosis is of primary concern^3^,^4^.

Viral infections have been associated with weak colonies and with colony collapse disorder of the Western honey bee (*Apis mellifera*)^5^,^6^,^7^,^8^,^9^,^10^. The most prevalent honey bee viruses have single-stranded RNA genomes and belong to species included in the families *Dicistroviridae* (*Acute bee paralysis virus*, ABPV; *Black queen cell virus*, BQCV; *Israeli acute paralysis virus*, IAPV; *Kashmir bee virus*, KBV) and *Iflaviridae* (*Deformed wing virus*, DWV; *Sacbrood bee virus*, SBV) in the order *Picornavirales*. Dicistrovirids and iflavirids have similar particle size of around 30 nm in diameter, preventing isolation of individual species from mixed infections by conventional methods^11^. Importantly, honey bee colonies are typically infected by multiple viruses^12^,^13^,^14^,^15^.

Due to the widespread infection of honey bees by multiple viruses, studies of individual virus species have been limited. Research focused on a single virus must account for potential background infections with multiple viruses in live bees. The outcome of mixed infections will depend on the level of interaction of each virus with the host and between viruses^16^, and the role of external biotic and abiotic factors^17^. For example, many honey bee viruses can covertly infect their host in chronic infections. However, a chronic infection can be switched to an acute, overt infection when a colony is infested with varroa mites (*Varroa destructor*)^9^. This ectoparasite spread to the Western honey bee from the Asian honey bee (*Apis cerana*) in the 1950s^18^ to the detriment of apiculture. Varroa mites feed on honey bee hemolymph thereby weakening the host, and inducing developmental and behavioral changes with consequences at both the individual and colony levels^18^. Added to this, the spread of the varroa mite has changed the impact of viral infections as the mite vectors multiple viruses (e.g. DWV^19^,^20^, ABPV^21^,^22^, IAPV^23^, SBV^14^ and KBV^24^; reviewed in^9^,^12^,^18^,^25^), enhances virulence in some cases^9^,^26^, and selects for particular virus strains^27^. Consequently, the coupling of infection with multiple viruses and high levels of varroosis can result in colony death^6^,^12^,^28^.

Further insight is needed into how the mixed virus infections of honey bees overcome the honey bee immune system to the detriment of colony health^4^,^29^. One approach to study virus-virus and virus-host interactions is the use of cell culture systems^11^. A bee-derived cell line would provide an aseptic system for the study of virus dynamics, allowing for synchronous infection of cells. Additionally, the use of a bee-derived cell line eliminates environmental variables that are impossible to control when working at the organismal and colony level; for example, the impact of the varroa mite in transmission of viruses and associated immunosuppression of bees.

Until recently, the lack of cell culture systems has restricted research in bee virology. Progress on the study of honey bee cell culture-virus interactions began with the establishment of a primary cell culture derived from the Asian honey bee. This primary culture was shown to support replication of Chinese sacbrood virus, CSBV, a strain of SBV^30^. Continuous bee cell lines, for example the line AmE-711 derived from embryonic tissues of the Western honey bee^31^ will significantly expand research options as seen for other insect viruses. For dicistrovirids in particular, the use of insect cell lines has been critical for important advances in understanding of infection at the molecular level. The DL2 and S2 cell lines derived from the vinegar fly (*Drosophila melanogaster*), have been critical for characterization of infection by cricket paralysis virus, CrPV, and Droshophila C virus, DCV, from deciphering viral entry mechanisms^32^ to description of specific sites of replication^33^, the study of viral suppressors of RNAi^34^,^35^ and cellular defense responses^36^,^37^. Undoubtedly, the investigation of molecular infection mechanisms of honey bee dicistrovirids and iflavirids will be favored by the use of established cell lines.

Based on the relevance of mixed virus infections in honey bees and their impact at both the individual and colony levels, we examined viral co-infections in honey bees and in the honey bee cell line AmE-711. This work highlights the potential use of a cell line for analysis of virus interactions of key importance to honey bee health.

## Results

### Infection of caged bees with a virus mixture rich in SBV results in IAPV-enriched infection

**Figure 1:**
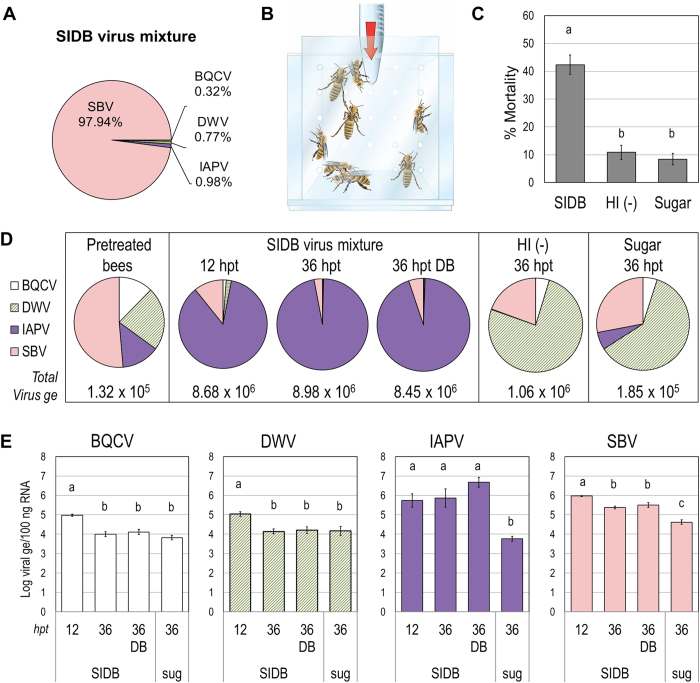
Viral dynamics in caged bees fed with a mixture of SBV-IAPV-DWV-BQCV (SIDB). (**A**) Proportion of each virus in the inoculum measured by RTqPCR. ABPV and KBV were below detection limits. (**B**) Bee inoculation; sucrose solution containing the test or control treatment was delivered in tubes at the top of the cage (arrow). (**C**) Average mortality in caged bees 3 days post-feeding in SIDB virus mixture in 30% sucrose solution, heat-inactivated particles (HI (−)) in sucrose solution or in sucrose solution alone (Sugar). Each bar represents 15 cages (+/− standard error of the mean). Letters denote significant differences between groups, Kruskal-Wallis ANOVA, Steel-Dwass posthoc test p < 0.05, Tukey HSD. (**D**) Average total virus loads (BQCV + DWV + IAPV + SBV genome equivalents (Virus ge) in 100 ng RNA) and proportion of each virus in different groups of bees. Pretreatment control bees are from 2 pools of 5 individual bees, HI virus treatment are from 3 pools of 3 cages (2 bees sampled/cage) whereas other samples represent 9 or 10 independent cages (2 bees sampled/cage). (**E**) Virus loads (log of viral genome equivalents (ge) in 100 ng of total RNA) analyzed by RTqPCR at different hours post-feeding. Bars represent average (+/− standard error of the mean) of pooled samples as indicated in (**D**). ABPV and KBV were below detection limits. DB, dead bees; hpt, hours post-treatment; sug, sucrose solution. Letters denote significant differences, BQCV, DWV, SBV: ANOVA, Tukey HSD, p < 0.05; IAPV: Kruskal-Wallis ANOVA, Steel-Dwass posthoc test, p < 0.05.

To analyze viral infection dynamics at the organismal level, we established a protocol to infect newly emerged bees by feeding. The virus inoculum was prepared by amplification of viruses by injection into bee pupae (see methods). Using a similar strategy, Boncristiani *et al.* (2013) obtained virus stocks composed mainly of IAPV. In our experience, the outcome of this protocol was variable, depending on the batch of pupae used, and stocks composed of just one virus were never obtained. For the present work, a mixture of unequal amounts of SBV-IAPV-DWV-BQCV (labeled “SIDB”), that was predominantly composed of SBV (Fig. 1A) was diluted in sugar solution and fed to caged bees (Fig. 1B). Sucrose solution either without virus or containing the SIDB inoculum inactivated by heat, were used as negative controls. Significant mortality was induced by the SIDB virus mixture at 3 days post-feeding compared to both the sucrose control and the heat-inactivated control (n = 15, Kruskal-Wallis ANOVA, H = 26.23, p < 0.00005; Steel-Dwass multiple comparison, p < 0.05; see Supplementary Table S1 for exact p-values of each comparison; Fig. 1C).

Because healthy, newly emerged bees can harbor low titers of different viruses, two pools of bees from the same batches used to prepare cages were sampled. Individual virus titers (genome equivalents or copies) were determined by one step reverse transcription-quantitative polymerase chain reaction (RTqPCR) in the pretreated bees, in inoculated bees 12 and 36 hours post-feeding (hpf) and in control-treated bees at 36 hpf. Initially, each virus was detected with independent standard curves but in order to make a valid comparison between different virus loads, a reference with all viral targets, a universal standard reference (USR) was designed (see Methods and Supplementary Fig. S1). An average total viral load of 1 × 10^5^ [sum of all detected virus (genome equivalents in 100 ng total RNA)], was detected in pretreated bees. This number increased in bees fed on the SIDB viral mixture to >8 × 10^6^ while control bees treated with heat-inactivated SIDB mixture or bees fed in sugar had lower viral loads (≤1 × 10^6^) after 36 hpf (Fig. 1D). Interestingly, when comparing the proportion of each virus in these bees, we found that IAPV was the dominant virus in infected bees despite the fact that SBV was the main component of the inoculum (Fig. 1D). This predominance of IAPV was not related to background virus profile as IAPV was present at levels similar to those of BQCV and DWV and at lower levels than SBV (Fig. 1A) and a homogeneous mixture of bees was used for all cages, regardless of treatment. In the negative control treatments (bees fed with sugar or heat-inactivated virus mixture), the predominant virus after 36 hours was DWV (Fig. 1D).

The BQCV load (log genome equivalents) was significantly higher in 12 hpf virus-treated bees than at other time points or in the control group (one-way ANOVA, F = 19.42, p < 0.05; Tukey HSD, p < 0.05, Fig. 1E). The same pattern was observed for DWV (one-way ANOVA, F = 6.29, p < 0.05; Tukey HSD, p < 0.05, Fig. 1E). For SBV, all virus-treated groups showed significantly higher titers than controls, but 12 hpf virus-treated bees showed significantly higher titers than both live and dead virus-treated bees at 36 hpf (one-way ANOVA, F = 33.7, p < 0.05; Tukey HSD, p < 0.05, Fig. 1E). IAPV levels were significantly higher in all virus-treated groups compared to sugar-fed controls, but there were no differences within the virus-fed groups (Kruskal-Wallis ANOVA, H = 20.93, p < 0.0005; Steel-Dwass multiple comparison, p < 0.05, Fig. 1E). Thus, virus inoculation resulted in increased BQCV and DWV levels 12 hpf, but these levels were no longer elevated at 36 hpf in either live or dead bees. SBV levels were elevated at all virus-treated time points, but were highest at 12 hpf, showing that SBV levels decreased with time. Only IAPV titers were elevated across all time points in virus fed bees compared to the control treatment.

### Infection of cultured cells with a virus mixture results in virus dynamics similar to those observed in young adult bees

**Figure 2:**
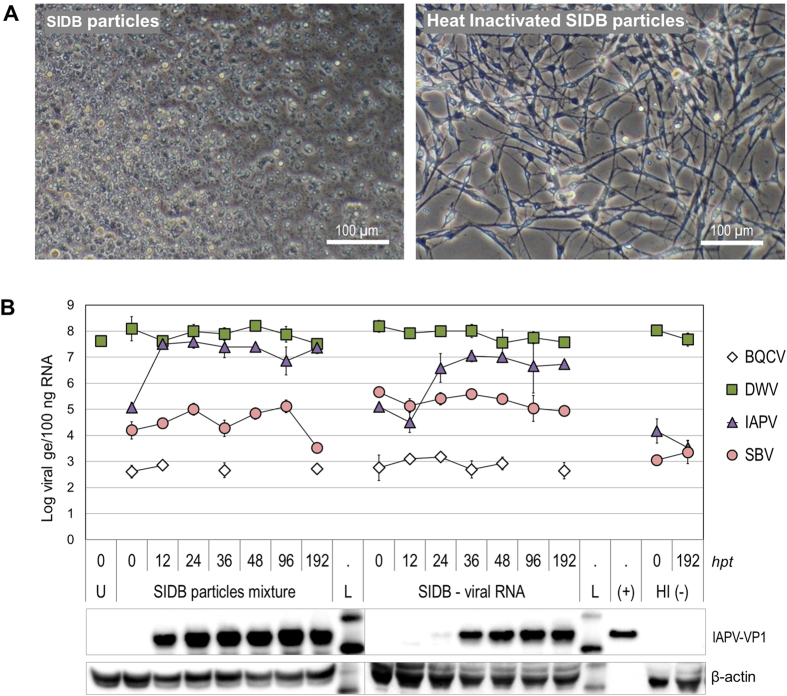
Viral dynamics in AmE-711 cells infected with SIDB. (**A**) Cells at 6 days post-transfection with SIBD or heat inactivated SIBD particles. (**B**) Virus loads (Log genome equivalents (ge) in 100 ng of total RNA) analyzed by RTqPCR at different hours post-treatment (hpt). Particle mixture, 4.7 ng of viral protein/well. Viral RNA, 50 ng of RNA/well. Bars represent average (+/− standard deviation) of 3 wells. Time 0 was taken 3.5 hours after treatment. Samples with BQCV loads below detection limit were not graphed. ABPV and KBV were not detected. Lower panel shows western blots to detect indicated proteins. U, untreated cells. L, protein molecular mass reference. (+), virion-enriched extract used as positive control for western blot.

**Figure 3:**
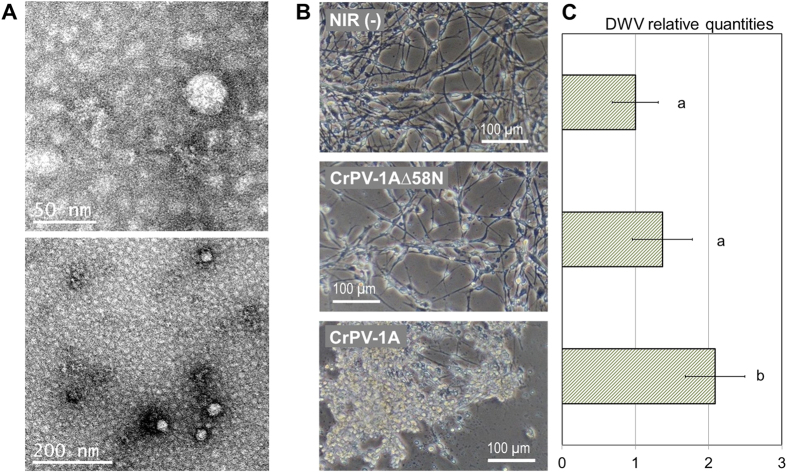
The AmE-711 cell line is persistently infected with DWV. (**A**) Virus-like particles from untreated AmE-711 cells observed under TEM. (**B**) Induction of CPE by CrPV-1A, a suppressor of RNAi. Images were taken 6 days post-treatment (dpt). (**C**) Relative quantities of DWV in cell treatments shown in B at 4 dpt. NIR, non-infective RNA (n = 5 wells) used as calibrator = 1 (corresponds to an average of 7.4 × 10^7^ DWV genome equivalents in 100 ng of total RNA); CrPV viral suppressor of RNAi, CrPV-1A (n = 3 wells); mutant viral suppressor of RNAi, CrPV-1A∆58N (n = 3 wells). Bars represent average replicates +/− 95% CI. Letters denote significant differences, ANOVA, Tukey HSD, p < 0.05. DWV levels were not significantly different at T0.

To study the dynamics of a mixed virus infection under more controlled conditions, we used the AmE-711 cell line derived from honey bee embryos^31^. Cells were inoculated with SIDB particles (Fig. 1A) or transfected with viral RNA extracted from virions (Supplementary Fig. S2). Cytopathic effects (CPEs) were visible 2 to 3 days post inoculation with the SIDB mixture. The typical fibroblast-like morphology of healthy cells gradually changed to detached, round cells and progressed until all cells in the well were floating and finally disintegrated (Fig. 2A and supplementary videos). The same cytopathic effects were observed when cells were inoculated with viral RNA, but not when treated with heat-inactivated particles (Fig. 2A) or non-infectious viral RNA (NIR, Fig. 3B).

Samples of treated cells were analyzed by RTqPCR over the course of 8 days to quantify the abundance of the four virus genomes. Additionally, the capsid protein VP1 of IAPV was detected by western blot. After 3–4 hours post-inoculation (or transfection), treatments were removed and the first sample was taken (T0); at this time, virus prevalence differed from the corresponding inoculum. In cells inoculated with particles, the dominant virus at T0 was DWV followed by IAPV, then SBV and BQCV. Differences between the viruses in the efficiency of early stage infection in AmE-711 cells could account for the rapid change in relative abundance (excluding DWV, see next section) from an SBV:IAPV ratio of 100:1 in the inoculum to 1:7 at T0 in the cells. For transfection of cells with viral RNA, the SBV:IAPV ratio of 3.8:1 at T0 was closer to the 5.4:1 ratio calculated for the inoculum (Supplementary Fig. S2). The difference in SBV:IAPV ratio between virions and viral RNA inocula can be explained by selective degradation of SBV RNA during RNA extraction from the virions: Over the course of this study, we also observed that SBV particles are more susceptible than those of other viruses in the SIDB mixture to degradation upon freeze-thawing (Fig. 1A, Supplementary Fig. S2).

By 12 hours post-treatment (hpt) we observed a rapid increase in IAPV genome equivalent levels by over two orders of magnitude in cells infected with SIDB particles. DWV was present at high levels from T0 until the end of the experiment; SBV increased by about 10-fold from T0 to 96 hpt and BQCV was barely detectable (Fig. 2B). When viral RNA was used as inoculum, an initial decrease in IAPV and SBV titers was observed, probably due to degradation of the RNA genomes by cellular RNases^38^. However, by 24 hpt, a substantial increase in IAPV was evident, as observed in cells inoculated with particles. IAPV-VP1 was detected by western blot in cells inoculated with viral particles and in cells inoculated with viral RNA (Fig. 2B).

IAPV replicated to high titers in AmE-711 cells compared to SBV, the main component of the SIDB inoculum. This result is not due to the inability of SBV to replicate in the cell line. In fact, an increase in SBV genome equivalents (ge) of >10 fold was evident after inoculation of cells with different dilutions of the SIDB mixture. When cells were treated with two dilutions of SIDB inoculum, SBV replicated to a maximum of 10^5^ genome equivalents/100 ng of total RNA (ge/100 ng RNA) at both dilutions: Starting from 3.8 × 10^4^ ge/100 ng RNA at T0 or from 7.3 × 10^3^ ge/100 ng RNA at T0. On the other hand, IAPV reached 10^7^ copies/100 ng total RNA regardless of the initial copies at T0 (2 × 10^5^ ge/100 ng RNA or <10^3^ ge/100 ng RNA; Supplementary Fig. S3).

### The AmE-711 cell line is persistently infected with deformed wing virus

During our experiments with the SIDB mixture, high quantities of DWV in the negative control groups were detected, including in untreated cells and cells inoculated with heat-inactivated particles (Fig. 2B). Tests for ABPV, BQCV, IAPV, KBV and SBV in untreated cells were all negative. The presence of DWV in the cells was corroborated with a second set of RTqPCR primers matching the 5′ half of the genome (Table S2). In addition, virus-like particles (VLPs) of the size of iflaviruses (30 nm in diameter) were observed in untreated cells (Fig. 3A). These particles are of the correct size for DWV, which along with our RTqPCR data, demonstrates that the cell line is persistently infected with DWV.

We amplified the full sequence of DWV from untreated cells in several fragments (see methods) that were assembled *in silico* to give a final sequence of 10,137 bases without the polyA tract (deposited in GenBank under accession number KT004425). The DWV-AmE711 isolate is 99% identical to DWV isolate PA (AY292384) with a total of 120 nt changes (Table S3). Moreover, the DWV-AmE711 polyprotein is 99% identical to that of DWV-PA. We did not find evidence for lethal mutations in the assembled sequence for the DWV-AmE711 isolate that would suggest impaired infectivity.

A model for maintenance of a persistent or covert state of insect RNA virus infection involves endogenization of viral sequences into the host genome via reverse-transcriptase activity and RNA interference (RNAi)^39^. We previously showed that the viral suppressor of RNAi (VSR), CrPV-1A^35^, was able to induce the acute infection of a persistent iflavirus in IPLB-LD-652Y, a cell line derived from the gypsy moth (*Lymantria dispar*)^40^. Similarly, this strong VSR induced CPEs in the AmE-711 cells persistently infected with a different iflavirus, DWV (Fig. 3B). The relative load of DWV following CrPV-1A treatment was more than twice the level of DWV in cells transfected with non-infectious viral RNA (NIR) (Fig. 3C). Cell damage was less severe when a mutant version of CrPV-1A missing the N-terminal 58 amino acids was transfected into the cells; and no CPEs were observed when NIR was used (Fig. 3B). The treatment with CrPV-1AΔ58N induced a 1.4-fold increase in the DWV load relative to the control treatment, but the DWV load was significantly lower than that induced by CrPV-1A (one-way ANOVA, F = 11.87, p < 0.05; Tukey HSD, p = 0.048, Fig. 3C). Nayak *et al.* (2010) reported that deletion of the C-terminal 40 residues renders CrPV-1A inactive^35^. Our results suggest that loss of the N-terminus impairs CrPV-1A VSR activity.

### IAPV dynamics on infection of cultured cells differ when KBV or SBV predominate in the inoculum

**Figure 4:**
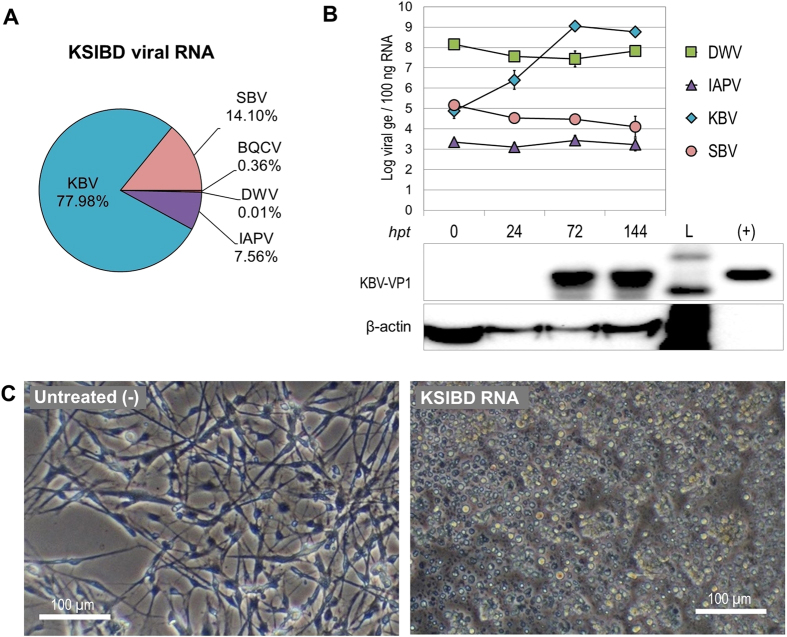
Viral dynamics in AmE-711 cells transfected with RNA from a virus mixture of KBV-SBV-IAPV-BQCV-DWV (KSIBD). (**A**) Proportion genomic RNA of each virus in the mixture used to transfect cells. ABPV was not detected. (**B**) Virus detection (Log genome equivalents (ge) in 100 ng of total RNA) in samples from cell culture transfected with 30 ng of KSIBD-RNA/well and collected at indicated hours post-treatment (hpt). Time 0 was 3.5 hours after transfection. BQCV was below detection limits. Each point represents the average and standard deviation of 3 wells. Lower panel shows western blot detection of indicated proteins. L, protein molecular mass reference. (+), virion-enriched extract. (**C**) Cells at 6 days post-transfection.

To investigate virus dynamics when SBV was not the predominant virus in the mixture used for inoculation, we obtained a sample rich in KBV (labeled “KSIBD”) derived from weak bee colonies from Wisconsin (Fig. 4A). We did not inoculate bee pupae or caged bees with the KSIBD mixture because of the limited quantity of inoculum, but was used directly for cell culture experiments to preserve the high levels of KBV in the inoculum (see methods).

Only 30 ng of viral RNA extracted from the KSIBD mixture was needed to infect AmE-711 cells, which was sufficient for analysis of virus dynamics (Fig. 4B) and induced strong CPEs (Fig. 4C). The genome equivalents of KBV increased dramatically and KBV-VP1 capsid protein was detected by western blot when KBV copies were >10^7^ ge/100 ng RNA, similar to the detection limit for IAPV-VP1 (Fig. 2B). There were no changes in total DWV loads over the course of the experiment with KSIBD, which is similar to the results for the SIDB mix (Fig. 2B). In addition, the loads of SBV decreased while IAPV remained stable (Fig. 4B). Interestingly, in this case IAPV did not reach the maximum copy number of 10^7^ ge/100 ng RNA observed in other experiments (Fig. 2B, S3).

## Discussion

This study provides the first comparison of *in vivo* (at the organismal level) and *in vitro* (in cell culture) infection by multiple honey bee viruses. The fact that the *in vivo* and *in vitro* studies resulted in similar virus dynamics, suggests that the study of viruses in cell lines provides results that are relevant to bee pathobiology. The AmE-711 cell line allows for research into the molecular dynamics of both acute and persistent infections by multiple honey bee viruses.

### Dynamics of mixed viral infection *in vivo*

The viral inoculum used for *in vivo* experiments resulted in significantly higher mortality than in the control groups, showing that this virus mixture caused predictable mortality *in vivo*. Despite the fact that mixed infections are likely to be the rule in nature rather than the exception, few systematic studies on co-infections have been conducted^16^. During our work with young bees, IAPV increased to a greater degree than SBV despite the high initial levels of SBV in the inoculum, suggesting that in some contexts IAPV has higher infectious capacity than SBV. The mechanisms behind this observation are unknown, but possibilities include differences between the viruses in virus entry and replication efficiency, competitive interactions, and in degradation rates. Additional factors to be considered for each virus are tissue tropism, maximum virus load capacity for susceptible tissues, life-stage specificity, and indirect interaction between viruses via the host immune system^16^,^17^.

Bailey reported that SBV was not transmissible by feeding to adult bees more than 4 days old, indicating that bees lose susceptibility to SBV infection as they mature^41^. The adult bees used in this work were less than 4 days old and hence susceptible to oral infection by SBV. We noted however that SBV was prone to degradation upon freeze-thawing, in agreement with the “rapid loss of infectivity of sacbrood virus anywhere except in the living host”^41^. We cannot rule out the possibility of a higher rate of SBV degradation in the inoculum during the feeding period compared to other viruses in the mixture (i.e. IAPV). It is notable however, that SBV was not completely degraded as SBV levels in infected bees were significantly higher than controls, even at 36 hpf.

Although the trends in experiments at the organismal level were clear and significant, viral loads were highly variable. The AmE-711 cell line allowed for testing of limited amounts of inoculum in a more controlled system than that offered by live bees, reducing the variability and allowing for more detailed observation.

### Dynamics of mixed viral infection *in vitro*

Similar to the results observed in young bees, IAPV replicated at higher levels than SBV in the cell culture system. This result was independent of the concentration of the inoculum, suggesting a differential maximum load or “saturation” limit for each virus in the system (persistently infected with DWV). Interestingly, this dynamic changed in the presence of high levels of KBV denoting complex dynamics and potential virus-virus interactions. At the cell level, if two or more co-infecting virus species require the same cellular machinery, the viruses will compete for cellular resources. For example, the internal ribosomal entry sites (IRESs) present on the genomes of all viruses in this study require a variety of ribosomal proteins to complete their infection cycle^42^. There is evidence that ribosomal proteins may be limiting factors for virus replication^43^. It is possible that IAPV and the very closely related KBV^12^ may compete more strongly for, or simply require fewer, ribosomal proteins than SBV or BQCV. With the KSIBD mixture, KBV gained over IAPV probably because they use similar machinery and KBV was more abundant from the start of infection. An additional consideration is that we did not monitor for KBV-IAPV recombination events which could impede accurate quantification of these viruses. Further studies are needed to determine why IAPV and KBV infect the bees and cultured cells so efficiently, to corroborate the ability of KBV to outcompete IAPV, and to determine scenarios for direct interaction (if any) between these viruses.

SBV was outcompeted by other viruses despite comprising almost 98% of the SIDB mixture. Initial preferential degradation of SBV in the inoculum and the absence of cell surface receptors for SBV in the AmE-711 cell line could partially explain the outcome, but once inside the cell, genome levels of the iflavirus SBV did not increase at the same rate as those of the dicistrovirids in genus *Aparavirus* (IAPV, KBV). Comparison between IAPV and SBV in the SIDB experiments and between KBV and SBV in the KSIBD experiments are warranted as similar starting loads were detected for these pairs at T0. In both cases the levels of SBV did not increase as dramatically as the levels of IAPV or KBV (that reached more than 2 orders of magnitude by 12–48 hpt). Another cell culture system, a primary cell line from the Asian honey bee has been reported to support replication of CSBV^30^. In that report, CSBV replication was detected by RTqPCR 2 days post-inoculation with a maximum of a 10-fold increase in virus load, which then rapidly declined. Factors that could limit SBV in this case are the lack of molecules needed for initial replication, suppression of virus replication by the RNAi pathway and inability of the virus to spread from cell to cell. Cell specialization may explain results for SBV in young bees with different pathogenicity at different developmental stages, i.e. SBV is reported to induce acute infections in larvae and pupae but persist in covert infections in adults^41^,^44^. Cell lines derived from different tissues are needed to address this concept. Another scenario is competitive exclusion whereby DWV in the cell line excluded or restricted SBV, for example by competing for cellular resources specific for iflavirids, as DWV did not exclude the aparaviruses IAPV and KBV.

### Challenges with the AmE-711 cell culture

As presented here, this system allows for molecular studies that will complement current tools used to investigate honey bee biology. The cell line also permitted screening for infectious clones of IAPV (Supplementary information, Supplementary Fig. S4). However, the use of this cell line was challenging as described below.

Our experimental design and biological replicates were limited by the availability of cells and by our inability to amplify the culture at Iowa State University, where cultures could not be maintained for more than 3 passages. Although living cells were maintained by diluting the original medium with fresh medium, the rate of cell division was very low (see supplementary movie file AmE-711-untreated) with a doubling rate of more than 7 days, rather than the reported 4 days^31^. A suitable batch of fetal bovine serum (FBS) may be the most critical component for successful maintenance of this cell line. Optimization to facilitate use of the cell line for in-depth studies of honey bee pathobiology is underway.

While no virus particles were observed in the AmE-711 cell line on preliminary examination by transmission electron microscopy (T.J. Kurtti, personal communication), we detected the DWV genome (using RTqPCR and sequencing) and particles (by TEM) in cells that were not inoculated. The origin of DWV in the AmE-711 cell line is unclear: Although cells could have been contaminated with DWV at some stage during establishment of the AmE-711 cell line, the prevalence of DWV suggests that embryonic cells used to develop the cell line were infected with DWV. DWV is widespread, and can infect honey bees chronically without inducing signs of infection. DWV is transmitted by mites, by direct contact between bees, and notably, vertically from queens and drones^19^,^45^,^46^,^47^. DWV has previously been detected in primary cultures of honey bee cells^48^, reinforcing the challenge that the high prevalence of DWV in honey bee colonies poses for procurement of virus-free tissues for establishing cell cultures.

Cytopathic effects were induced without a dramatic increase in DWV levels after CrPV-1A treatment of AmE-711 cells. This result may be explained by the cells being close to a hypothetical maximum viral RNA load, and a 2-fold change was sufficient for induction of CPE. Another possibility is that CrPV-1A is affecting cellular pathways other than the siRNA pathway. Nayak *et al.* (2010) reported that CrPV-1A does not interfere with the miRNA pathway, but recent work shows that CrPV-1A also inhibits silencing via the miRNA pathway in S2 cells^49^. No DWV-free cell line was available to test whether CrPV-1A alone could induce CPEs in honey bee cells; how CrPV-1A leads to AmE-711 cell death requires further investigation.

The presence of DWV may increase the sensitivity of AmE-711 cells to changes in culture medium and culture conditions, with stress resulting in acute infection and cell death. Biotic and abiotic stresses can induce DWV to switch from chronic to acute infection^50^. The titer of DWV increased when honey bees were under abiotic stress such as temperature change^51^, exposure to insecticides^52^, or acaricides^53^, or biotic stress such as coinfection with other viruses, microsporidian pathogens (*Nosema sp*), and mite infestation^28^,^54^,^55^. We propose that this cell line can be used as a model that mimics honey bee processes at the organismal level and that the cell line can be used to study biotic and abiotic factors that induce acute infection of DWV. This cell line will also help to uncouple the impact of varroa mite parasitism and DWV infection at the cellular level, and could eventually provide a clean source of DWV particles for additional analyses.

## Conclusions

The following observations from the current study provide several avenues for further investigation: 1. Virus infection dynamics will be determined by the context of infection, as demonstrated by the dynamics of IAPV in two different virus mixtures. 2. Virus-virus interactions within the honey bee pathosystem may occur directly through interaction of viral products and competition for cellular resources or indirectly via the anti-viral defenses and physiological response of the host. 3. Superinfection of honey bee cells is shown here for the first time. We assume that practically all AmE-711 cells are infected with DWV although *in situ* localization is needed to corroborate this. The presence of DWV did not prevent accumulation of IAPV or KBV to high levels, sufficient to induce cell death. 4. DWV persistently infects the AmE-711 cell line, mimicking chronic infection by this virus at the individual bee and colony levels. On addition of the heterologous strong suppressor of RNAi, CrPV-1A, DWV replication increased resulting in acute infection and cell death. This result highlights the potential for indirect interaction between viruses via the RNAi pathway. 5. Virus dynamics in the AmE-711 cell line resembled those observed in young adult bees. The cell line allowed for more detailed analysis of virus dynamics under more controlled conditions. Starting from similar loads of SBV- IAPV or SBV- KBV, iflavirid SBV replicated at lower levels than the dicistrovirids. The AmE-711 cell line and other bee cell culture systems will allow for molecular analysis of virus-virus and virus-host cell interactions for further study of factors that modulate infection.

## Methods

### Virus inoculum

Virus mixtures were amplified in pupae as previously reported^56^,^57^ with some modifications. First, 50 virus-infected adult bees were homogenized in 100 mL 1X PBS buffer. Debris was eliminated by two centrifugation steps (15,000 × g for 5 min) and viral particles were enriched by 7% PEG 8000–2.3% NaCl precipitation^58^. Viral particles were resuspended in 8 mL TES (10 mM Tris-HCl pH 7.5, 2 mM EDTA, 150 mM NaCl) and concentrated to 1 mL using Microcon centrifugal units 100k (Millipore). Pupae at the white eye stage were removed carefully from the combs and microinjected (Harvard Apparatus Model PLI-100 Pico Injector) with 1 μL of viral particles extracted from infected adult bees. Infected pupae were incubated at 32 °C for 5–7 days and then collected and stored at −70 °C. This material was used to enrich particles as described above but with an additional extraction with 0.3 volumes chloroform:isoamyl alcohol (24:1) after elimination of debris. In addition, final particles were cleaned twice by passage through a cushion of 20% sucrose/TES (w/v) (24,000 × g at 4 °C for 4 hours). Virus titers were amplified more than 4 orders of magnitude using this pupal injection protocol. The process was repeated using different groups of approximately 500 pupae. Viral particle stocks were diluted in TES (approximately 0.5 mL for 50 initial bees) and stored at −70 or −20 °C until use. The final SIDB mixture was the sum of different stocks and the total protein in the non-diluted composite was 4.7 mg/mL. Particles were passed through a 0.2 μm filter before use for cell infection. An aliquot of viral particles inactivated by heating at 95 °C for ≥30 min was used as a negative control treatment for infection experiments of AmE-711 cells and young adult bees. SIDB viral RNA was extracted from 100 μL of stock particles with 1 mL TRIzol following the supplier’s protocol (Life Technologies) except for initial incubation at 65 °C for 10 min. The KSIBD viral mixture was obtained from particles extracted from adult bees without any further amplification in pupae. KSIBD viral RNA was extracted with TRIzol as described and used to transfect cells. The proportion and amount (ge/100 ng RNA) of ABPV, BQCV, DWV, IAPV, KBV and SBV in the final viral RNA from each stock was obtained by RTqPCR as described below. To calculate the percentage of each virus in virion preparations, the SIDB stock was diluted 1:100 and used directly for RTqPCR. Isolation of individual virus species was not attempted. The proportion of each virus in virion stocks was variable resulting in part from the composition of virus used for pupal injection.

### Infection of caged bees

Frames of emerging brood were removed from 15 healthy colonies from the research apiary at Iowa State University and kept overnight in emergence boxes in an incubator at 32 °C and 50% relative humidity. The next day, newly-emerged bees from these frames were mixed together and pools of 10 randomly selected bees were frozen at −70 °C to determine background virus loads in the bees before treatment. Remaining bees were placed inside clear acrylic cages (10.16 cm × 10.16 cm × 7.62 cm) in groups of 35 per cage. Cages were housed in an insect rearing room at 32 °C and 50% relative humidity for the entire experiment. To treat the caged bees, the virus particle stock SIDB was diluted 1:1000 in 0.6 mL 30% sucrose solution. This dilution was calculated based on preliminary LD50 analyses. Each cage received an open feeder containing either SIDB in 0.6 mL 30% sucrose solution, heat-inactivated SIDB in sucrose solution, or sucrose solution alone. Bees had *ad libitum* access to the feeder for 12 hours, during which the solution was completely consumed. Bees then received *ad libitum* access to untreated sucrose solution for the remainder of the experiment. This design was repeated twice, once with 7 cages/treatment and once with 8 cages/treatment, resulting in a total of 15 cages/treatment. Mortality was monitored in all cages every 12 hours and any dead bees were collected and stored at −70 °C. Pools of two live bees were collected from each cage at 12, 36, and 60 hours post-treatment (when the experiment was terminated) and stored at −70 °C until RNA extraction. For virus quantification, 9–10 cages (4 or 5 from each replicate) were randomly chosen for analyses

### Cell culture

AmE-711 cells (provided by University of Minnesota) were maintained at 32 °C in HB-1 medium supplemented with 10% FBS (Biowhittaker or Sigma-Aldrich) and 5% tryptose phosphate broth as described^31^. AmE-711 cultures seeded in 25 cm^2^ polystyrene flasks (Falcon) were maintained by replacing 25–50% of the medium every 3 days with fresh medium.

### DWV-AmE711 isolation and sequencing

DWV was extracted from untreated AmE-711 cells by a slow centrifugation protocol^59^. The genome of the DWV-AmE711 isolate was amplified by two-step RT- PCR from enriched particles using multiple primers (Table S6) designed based on the sequence of DWV-PA isolate (GenBank AY292384). Several overlapping fragments were sequenced to assemble the final genome using DNA Dynamo software (Blue Tractor Software). The 5′ and 3′ end sequences were not corroborated by any other technique. The final 10,137 base sequence was deposited in the GenBank database with accession number KT004425.

### *In vitro* transcription

DNA coding for the wild type CrPV-1A viral suppressor of RNA silencing was amplified from CrPV-1A-TOPO plasmid^40^ using primers Cr1AT7F and Cr1ARev (Table S4). A mutant version missing 58 amino acids at the N-terminus was generated by PCR amplification of the same DNA with primers Cr1AT7∆N-F and Cr1ARev (Table S4). Final templates were cleaned by phenol extraction and sequenced. 5′ capped and 3′ polyadenylated *in vitro* transcripts were prepared using T7 mScript kit (CellScript) followed by phenol extraction as recommended by the manufacturer. Negative controls of non-infective RNA (NIR, Fig. 3B) were *in vitro* transcripts synthesized from an aphid lethal paralysis virus (ALPV) amplicon and IAPV cloned into the pJazz vector (Lucigen) (Supplementary Fig. S4 and Supplementary Information). The IAPV clone included an A25 sequence at the 3′ end resulting from a reverse primer that included a T25 sequence (Table S2).The resulting IAPV transcript was capped at the 5′ end and the 3′ A25 tail was extended with T7 mScript kit (CellScript).

### Cell treatments

AmE-711 cells that were ≥80% confluent were trypsinized using 0.25% Trypsin-EDTA (Life Technologies). Dissociated cells were then seeded into wells of 24-well polystyrene plates (Falcon) at a density of 10^5^ cells/well in 400 μL complete medium, and incubated at 28 °C. After 3 days, when cells showed characteristic fibroblast-like extensions, complete medium was replaced with 200 μL serum-free Leibovitz’s L-15 Medium (Life Technologies). Cells were inoculated either by mixing 1 μL of viral particles with 60 μL of L-15 medium per well, or by transfection with 1 μL of RNA [50 ng viral RNA or NIR, or 1 μg CrPV-1A wt or mutant CrPV-1A] using 3 μL of Cellfectin^®^ II Reagent (Life Technologies) in a final volume of 60 μL of L-15 medium per well, following the supplier’s protocol. After 3.5–4.5 hours of slow agitation at room temperature treatments were removed, complete medium was added and the cells were incubated at 28 °C until sampling. The replicates (≥2 wells) of each treatment per time point were sampled by scraping cells and collecting the entire contents of the well. Samples were stored at −20 °C until RNA/protein extraction.

### RTqPCR

Total RNA was extracted from honey bee cell samples or individual bees using TRIzol LS or TRIzol (Life Technologies) respectively. Fragments of 90–200 bp of viral genomes (Table S2) were amplified in one step RTqPCR using the iTaq™ Universal SYBR^®^ Green One-Step Kit (BioRad) following an absolute quantification approach^60^ using 100 ng of total RNA per sample. Amplification with technical duplicates was performed in a CFX 384 thermocycler (BioRad) programmed as follows: Reverse transcription [50 °C - 25 min] – PCR [95 °C - 5 min] - 40 cycles of [95 °C - 5 sec, 58 °C - 30 s] – Melting curve [95 °C - 30 s, 55 °C - 30 s] – [stepwise 0.5 °C increases (10 s/step) from 55–95 °C]. The final copy number (ge/100 ng RNA) of each virus in unknown samples was calculated by extrapolation to a standard curve made by serial dilution (1:10) of a viral fragment RNA used as reference. These virus reference sequences were generated by RT-PCR from positively infected bee samples, cloned in pCR4-TOPO vector (Life Technologies) and confirmed by sequencing. Plasmids were linearized or used as PCR template to generate a T7-DNA template for *in vitro* transcription. *In vitro* transcripts of each viral fragment were prepared with the MegaScript kit (Life Technologies) followed by DNAseI treatment. The final virus reference RNA was quantified in a Qubit fluorometer (Life Technologies) and copy number calculated based on sequence [ http://www.endmemo.com/bio/dnacopynum.php]. Specific primers for each virus are listed online in Table S2. In the case of IAPV and SBV, a second set of primers was used to confirm results (Table S2). To corroborate results from individual references and accurately compare copy numbers of different viruses, we synthesized a DNA template (GenScript) containing all relevant viral amplicon sequences (universal standard reference, USR) concatenated under a T7 promoter and cloned in pUC57-Kan vector (Supplementary Fig. S1). The plasmid was linearized with XbaI and cleaned by phenol extraction. The final USR-RNA reference was prepared as described for each separate virus. Different amplification efficiencies with primers used for different viruses was observed when using the USR (equal copy numbers are expected if all primers have equal efficiency since the reference is the same). Individual genome equivalents or viral copies were calculated using the CFX program. Quantification analyses to compare abundance between viruses were done in qBase + software considering individual target efficiencies. Data out of the dynamic range for each target were considered below detection limits (Table S5).

### Western blot

A rabbit antibody directed to an epitope conserved in the VP1 of IAPV, KBV and ABPV (GGRRYKFFNTTPLK) was synthesized by GenScript. Total protein was extracted with TRIzol from cell culture samples (after RNA extraction) and diluted in 1% SDS following the supplier’s protocol. Equivalent amounts of protein from corresponding replicates of each sample were pooled and 50 μg were separated in 4–12% NuPAGE Bis Tris precast gels (Life Technologies). Proteins were blotted to Immun-Blot LF PVDF membrane (BioRad) and probed with rabbit anti-beta actin (0.5 μg/mL) (NeoBiolab) and then with a secondary anti-rabbit IgG (0.1 μg/mL) conjugated to HRP (GenScript). The signal was detected using Immun-StarWesternC kit (BioRad) in a ChemiDoc XRS + system (BioRad). Membranes were stripped using Western Reprobe PLUS buffer (BioSciences) for 1 hour at room temperature and then reprobed with rabbit anti-VP1 (1 μg/mL) followed by incubation with anti-rabbit IgG and detection as described before.

### Transmission Electron Microscopy

Viral particles were obtained from untreated cells by slow centrifugation as reported^59^. Three μL of particles were stained with 3 μL of 2% uranyl acetate in a carbon grid. Particles were detected with a JEM 2100 transmission electron microscope at the Microscopy and NanoImaging Facility at Iowa State University.

### Statistical analyses

All datasets were screened for normality using normal probability plots and homogeneity of variance was tested using the Levene’s test (alpha value p > 0.05). Honey bee cage mortality data (n = 15 per group) were not normally distributed, so data were tested using a nonparametric Kruskal-Wallis ANOVA followed by a Steel-Dwass posthoc multiple comparison with an alpha value of 0.05.

To evaluate virus titers in cages bees, a subset of the 15 cages from each group were randomly selected for analysis. Here, we compare BQCV, DWV, SBV, and IAPV titers between live, virus-treated bees at 12 hpi (n = 9) and 36 hpi (n = 10), virus-treated dead bees at 36 hpi (n = 9), and live, sugar-control bees at 36 hpi (n = 10). Virus titers in caged bees (viral genome equivalents in 100 ng of total RNA) were log-transformed before statistical analyses^57^. For BQCV, DWV, and SBV, viral loads in caged bees were compared across time points using a one-way ANOVA followed by a Tukey HSD posthoc multiple comparison and an alpha value of 0.05. As IAPV data did not fit the assumption of homogeneity of variance, these data were tested using a nonparametric Kruskal-Wallis ANOVA followed by a Steel-Dwass posthoc multiple comparison.

To evaluate differences in relative DWV titer in cells treated with CrPV-1A (n = 3), mutant CrPV-1A (n = 3) and non-infectious RNA (n = 5), a one way ANOVA was followed by a Tukey HSD posthoc multiple comparison and an alpha value of 0.05.

These analyses were performed using the statistical software JMP Pro v.11. For test statistics and exact p-values for each comparison, see Supplementary Table 1.

## Additional Information

**Accession codes:** The sequence of DWV-AmE711 was deposited in GenBank under accession number KT004425.

**How to cite this article**: Carrillo-Tripp, J. *et al.* *In vivo* and *in vitro* infection dynamics of honey bee viruses. *Sci. Rep.* **6**, 22265; doi: 10.1038/srep22265 (2016).

## Supplementary Material

Supplementary Information

Supplementary Video 1

Supplementary Video 2
