# Supplementary material for: In vivo and in vitro infection dynamics of honey bee viruses
Source: Sci Rep. 2016 Feb 29;6:22265. doi: 10.1038/srep22265 (PMC4770293; doi:10.1038/srep22265)
Supplement: Supplementary Information [file srep22265-s1.pdf]

***In vivo* and *in vitro* infection dynamics of honey bee viruses**

Jimena Carrillo-Tripp[1, 2], Adam G. Dolezal[1], Michael J. Goblirsch[4], W. Allen Miller[2], Amy L. Toth[1,3], Bryony C. Bonning[3*]

1. Department of Ecology, Evolution, and Organismal Biology, Iowa State University, Ames, IA 50011, USA
2. Department of Plant Pathology and Microbiology, Iowa State University, Ames, IA 50011, USA
3. Department of Entomology, Iowa State University, Ames, IA 50011, USA
4. Department of Entomology, University of Minnesota, St. Paul, MN 55108, USA
* Corresponding author

Supplementary FIGURES

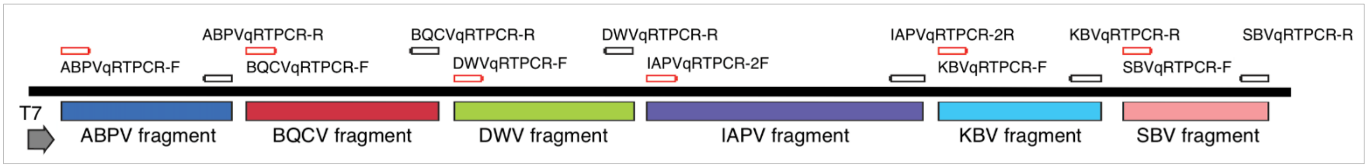

Fig. S1. Representation of DNA template used to synthesize the Universal Standard Reference (USR) for viral quantification by RTqPCR. The DNA was synthesized and cloned into pUC57-Kan (not depicted) by GenScript. Primer sequences are listed in Table S2. (Figure made in DNA Dynamo and Adobe Photoshop).

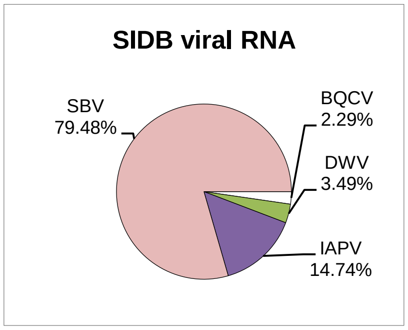

Fig. S2. Viral RNA extracted from SIDB particles (after several rounds of freezing/thawing) used to transfect cells.

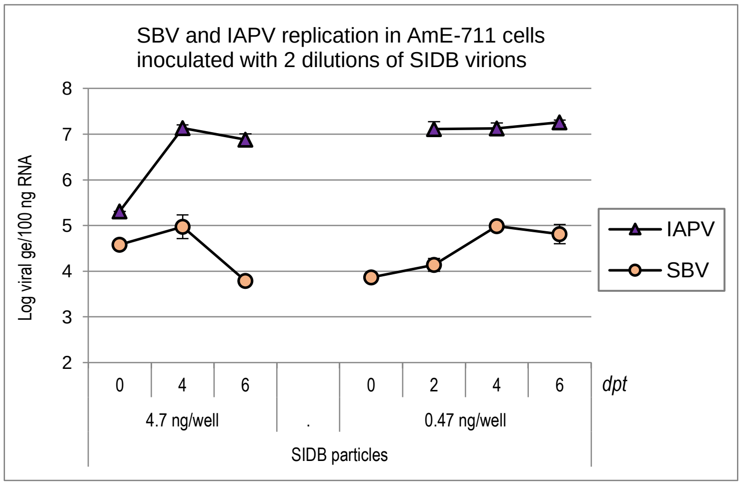

Fig. S3. SBV can replicate in AmE-711 cells. Cells infected with 2 dilutions of SIDB particles were analyzed by RTqPCR to determine viral loads (genome equivalents, ge) at several days post-treatment (dpt). Bars represent average (+/- standard deviation) of 2 wells. Time 0 was taken after 4.5 hours of treatment. At T0, in cells treated with

0.47 ng of viral protein/well, IAPV was below the detection limit as was one replicate of SBV. Each virus load was calculated with the corresponding individual standard reference. Inoculation with viral particles was mediated by transfection with cellfectin in this experiment.

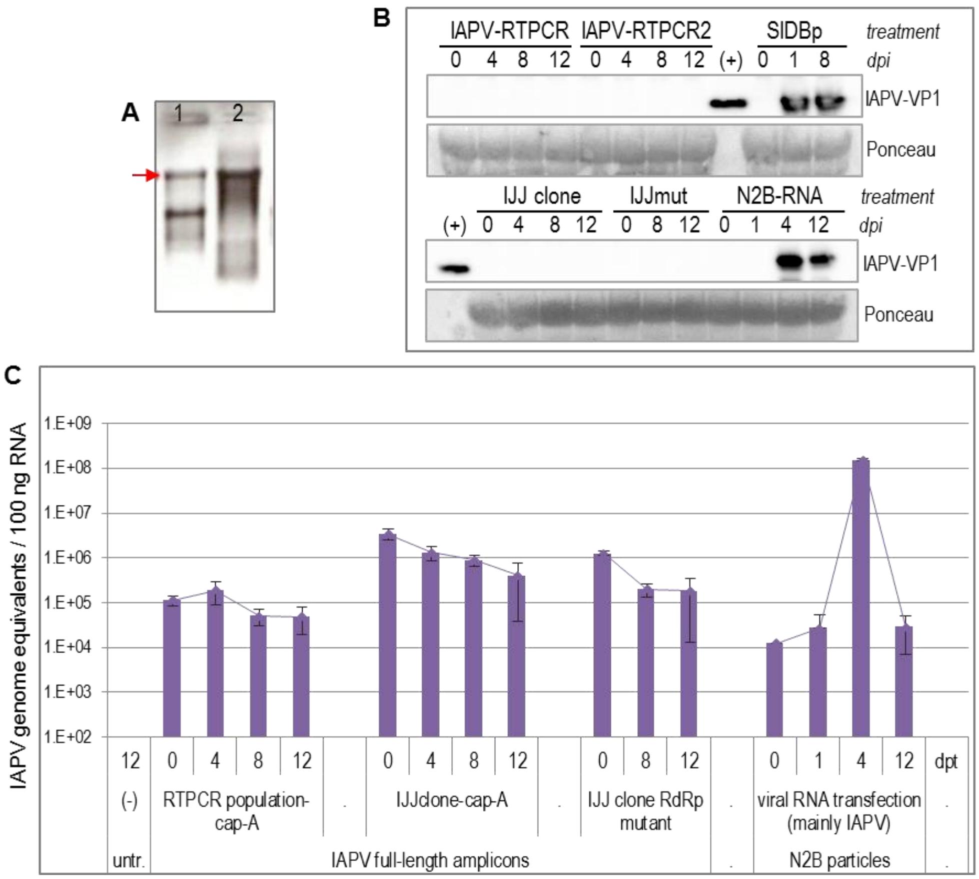

Fig. S4. AmE-711 transfection with full-length IAPV *in vitro* transcripts. A. Lane 1, *in vitro* transcript from full-length IAPV amplicon (IAPV-RTPCR). Lane 2, viral RNA extracted from particles. Arrow shows the >9.5 kb bands corresponding to full-length viral genome. B. IAPV-VP1 protein detection by western blot. SIDBp, sample from cells treated with the mixture of particles as described in the main text. (+), enriched SIDB particles used as positive control for VP1 detection. IAPV-RTPCR and IAPV-RTPCR2, samples from cells treated with amplicon-derived transcripts uncapped and capped-polyadenylated respectively. dpi, days post inoculation. (-), untreated cells. N2B particles, mixture composed of 99.9% IAPV. IJJ clone, IAPV in pJazz. IJJ clone RdRP mutant (insertion in coordinate 5,296) gives a C-terminal deletion of non-structural polyprotein in the RdRp coding region. C. IAPV genome equivalents in 100 ng total RNA measured by RTqPCR. All IAPV full-length amplicons were transcribed *in vitro*, polyadenylated and capped. Cells were transfected using 50 ng of *in vitro* RNA per well or 5 ng of viral RNA/well. T0 samples were taken once medium was changed at 3.5 hours after treatment.

Supplementary TABLES

Table S1. Statistical analyses

**Comparison of mortality of caged bees**		
Kruskal-Wallis, H=26.23; Steel-Dwass posthoc multiple comparison		
**Comparison**	**Z**	**p**
SIDB:Sugar	4.44	8.96885E-06
SIDB:HI(-)	4.31	0.000015845
Sugar:HI(-)	-0.48	0.632606

**Comparison of BQCV titers in caged bees**	
One-way ANOVA, F=19.42, p=1.63e-7; Tukey HSD posthoc comparison	
**Comparison**	**p**
SIDB 12 hpt: sugar 36 hpt	0.00000021
SIDB 12 hpt: SIDB 36 hpt	0.0000046
SIDB 12 hpt: SIDB 36 hpt dead	0.000056
SIDB 36 hpt dead: sugar 36 hpt	0.32
SIDB 36 hpt: sugar 36 hpt	0.71
SIDB 36 hpt dead: SIDB 36 hpt	0.89
**Comparison of DWV titers in caged bees**	
One-way ANOVA, F=6.29, p=0.0016; Tukey HSD posthoc comparison	
**Comparison**	**p**
SIDB 12 hpt: sugar 36 hpt	0.0039
SIDB 12 hpt: SIDB 36 hpt	0.0049
SIDB 12 hpt: SIDB 36 hpt dead	0.0098
SIDB 36 hpt dead: sugar 36 hpt	0.994
SIDB 36 hpt: sugar 36 hpt	0.9981
SIDB 36 hpt dead: SIDB 36 hpt	0.9998
**Comparison of SBV titers in caged bees**	
One-way ANOVA, F=33.7, p=2.7e-10; Tukey HSD posthoc comparison	
**Comparison**	**p**
SIDB 12 hpt: sugar 36 hpt	1E-10
SIDB 12 hpt: SIDB 36 hpt	0.0000012
SIDB 12 hpt: SIDB 36 hpt dead	0.000015
SIDB 36 hpt dead: sugar 36 hpt	0.00067
SIDB 36 hpt: sugar 36 hpt	0.013
SIDB 36 hpt dead: SIDB 36 hpt	0.75

Table S1 continued

Comparison of IAPV titers in caged bees		
Kruskal-Wallis ANOVA, H=20.93; Steel-Dwass posthoc comparison		
**Comparison**	**Z**	**p**
SIDB 12 hpt: sugar 36 hpt	3.63	0.0016
SIDB 12 hpt: SIDB 36 hpt	-0.12	0.99
SIDB 12 hpt: SIDB 36 hpt dead	1.32	0.54
SIDB 36 hpt dead: sugar 36 hpt	3.55	0.0022
SIDB 36 hpt: sugar 36 hpt	3.44	0.0033
SIDB 36 hpt dead: SIDB 36 hpt	0.77	0.86

Comparison of DWV titers in AmE-711 cell culture	
One-way ANOVA, F=11.87, p=0.004; Tukey HSD posthoc comparison	
**Comparison**	**p**
NIR: CrPV-1AΔ58N	0.2798
NIR: CrPV-1A	0.0031
CrPV-1A: CrPV-1AΔ58N	0.0486

Table S2. Virus-specific primers used in this study.

Virus	Primer	Sequence 5' – 3'	Product size (bp)	Product Tm (°C)	Reference
Acute bee paralysis virus	ABPVqRTPCR-F	ACCGACAAAGGGTATGATGC	124	79	1
	ABPVqRTPCR-R	CTTGAGTTTGCGGTGTTCCT			
Black queen cell virus	BQCVqRTPCR-F	TTTAGAGCGAATTCGGAAACA	140	79	
	BQCVqRTPCR-R	GGCGTACCGATAAAGATGGA			
Deformed wing virus	DWVqRTPCR-F	GAGATTGAAGCGCATGAACA	130	80	
	DWVqRTPCR-R	TGAATTCAGTGTCGCCCATA			
Sacbrood bee virus	SBVqRTPCR-F	GGGTCGAGTGGTACTGGAAA	105	77	
	SBVqRTPCR-R	ACACAACACTCGTGGGTGAC			
Kashmir bee virus	KBVqRTPCR-F	TGGCATCGAGCGCATTCCAG	118	81.5	This work
	KBVqRTPCR-R	TCGGGGTTTGGACCACCCGAAT			
Israeli acute paralysis virus	IAPVqRTPCR-2F	GCACAGTCTTCTGGTGATTGC	200	80.5	
	IAPVqRTPCR-2R	GTTAGCACACGATTGGTTATCAGC			
Deformed wing virus *	DWVqRTPCR-2F	CCTGATGGCGAGGGTGAAG	100	77.5	
	DWVqRTPCR-2R	TTACGCTCACCGGCGCT			
Israeli acute paralysis virus *	IAPVqRTPCR-F	TGCAAGTGAACGCCCCAAAAACG	197	80	
	IAPVqRTPCR-R	TGCCACAGTTCCGACAACATCTGC			
Sacbrood bee virus *	SBVqRTPCR-2F	AAGTGTGGATCACCTAGTGG	94	75	
	SBVqRTPCR-2R	CTACTAACGTCTCCCAAGCTACG			
Israeli acute paralysis virus (full genome) *	IAPV-BamT7	actactGGATCCTAATACGACTCACTATAgCGTACAATTTTCGCCGAAATTAGC	9,647	N/A	
	IAPV-BamT25	actactGGATCCtttttttttttttttttttttttttGAAATTTACCTAATTCGAAAATTTTGCCCACTCTACC			

* These primers do not recognize the USR shown in Fig. S1

Table S3. Summary of nucleotide differences between DWV-AmE-711 KT004425 (10,137 b) and DWV-PA AY292384.1 (10,135 b).

Region	Type	Changes
Untranslated regions	Substitution	15
	Insertion	2
Polyprotein	Silent	74
	Conservative	18
	Missense	11
	Total	120

Table S4. Primers used for CrPV-1A constructs.

Primer	Sequence	Reference
Cr1AT7F	tAGATCTTAATACGACTCACTATAGCGCCACCATGTCTTTTCAACAAACAAACAAC	2
Cr1ARev	atGCGGCCGCGTTGCGTTCTCTTTCACAAGAAG	
Cr1AT7ΔN-F	tAGATCTTAATACGACTCACTATAGCGCCACCgatTCTTTTCAAC	This work

Table S5. USR efficiency and dynamic range for each primer set. Values smaller than the corresponding low Cq limit were considered below detection limits.

	Efficiency %	Slope	Y-Intercept	R^2	Dynamic Range with Threshold=1000 RFU			
					Low Cq Mean	Calculated Copies	High Cq Mean	Calculated Copies
ABPV	60.24	-4.884	50.641	0.978	5.54	4.92E+08	33.72	4.92E+02
BQCV	60.33	-4.878	49.775	0.985	6.13	4.92E+08	35.76	4.92E+02
DWV	58.67	-4.988	51.287	0.987	6.88	4.92E+08	31.54	4.92E+03
IAPV	54.62	-5.283	52.445	0.966	4.84	4.92E+08	36.75	4.92E+02
KBV	56.38	-5.15	56.756	0.98	6.68	4.92E+09	35.16	4.92E+03
SBV	54.76	-5.273	53.948	0.987	1.96	4.92E+09	32.17	4.92E+03

Table S6. DWV primers used for sequencing of DWV-AmE-711.

Primer	Sequence	Reference
DWV-1F	CGATTTATGCCTTCCATAGCGAATTAC	This work
DWV-1793F	CCTGATGGCGAGGGTGAAG	
DWV-1560F	GCAACTCGCTTCGTTCGTG	
DWV-2404F	CCTTACGGATGAGTGCTAC	
DWV-3846R	CTCCTTCTGGAATAGCCTC	
DWV-4045F	CCACAAGGGTTAGCGTTAGAC	
DWV-4230R	CGATCCGGTCGATGTTGTACC	
DWV-5617R	CTCGCACGTAAGAGCTCG	
DWV-661R	CGAATCACCTCTAAACTAG	
DWV-7062F	GCATGGTTGTTCGAGAACC	
DWV-7774F	GATTGTATTATGGTGGTCTCG	
DWV-6249F	ATCAGCGCTTAGTGGAGGAA	3
DWV-1893R	TTACGCTCACCGGCGCT	This work
DWV-6470F	GAGATTGAAGCGCATGAACA	1
DWV-6600R	TGAATTCAGTGTCGCCCATA	
DWV-503F	CCACTGCAGTATCGAGTAGAG	This work
DWV-1178R	GACGGCAGAGTAAGAAAGAGTTCC	
DWV-8656F	CACCTGGAACATCGGGTAAG	4
DWV-9356R	AGAGAACTCGGACAAAGGC	
DWV-T15-10135R	TTTTTTTTTTTTTTTACTATTATGGTTAAAACTATAC	This work

Supplementary MOVIES

File AmE-711-SIDBvirusmix.mov: Movie of AmE-711 cells infected with SIDB particles
File AmE-711-untreated.mov: Movie of untreated AmE-711 cells.

Supplementary INFORMATION

Dicistrovirus full-length amplicons

An infectious clone of CrPV was recently reported [5]. CrPV is the type member of the genus *Cripaviridae*. Based on the demonstrated infectivity of uncoated IAPV viral RNA (Fig. 2B), we followed two strategies to obtain *in vitro*-produced IAPV infectious RNA. IAPV is a member of the sister genus *Aparavirus* in the family *Dicistroviridae*.

In the first method, the full-length sequence of IAPV was cloned under a T7 promoter (IJJ clone). The second strategy was to use T7-IAPV non-cloned amplicons to preserve viral population diversity (IAPV-RTPCR). Gel electrophoresis of *in vitro*-produced transcripts from clones and amplicons included a band that was smaller than the full genome (Fig. S4A), most probably a product of a cryptic T7 promoter, or a termination signal in the IAPV genome. These transcripts were used to transfect AmE-711 cells. Viral RNA extracted from a virus preparation composed mainly of IAPV (labeled N2B) was used as positive control.

Cells treated with N2B viral RNA showed signs of infection after 3 dpt; at 12 dpt just floating material was present in the wells. In contrast, cells treated with IAPV clones or amplicons *in vitro* RNA showed signs of stress but most remained attached to the well and, although viral RNA was detected by RTqPCR after one week, there was no increase in viral loads or capsid protein VP1 (Fig S4B-C), suggesting that the clones or amplicons were unable to infect AmE-711 cells under the conditions tested.

More work is needed to generate IAPV infectious clones and to unravel the potential interference of the extra band (<9.6 kb) in our inocula. The AmE-711 cell line, or new lines, will facilitate the testing of different strategies for development of infectious clones of honey bee viruses [6].

Supplementary METHODS

Full virus genome amplification and cloning

The full-length genome of the aphid lethal paralysis virus (ALPV) was amplified in one PCR reaction after reverse transcription of viral RNA as previously described [7]. A similar strategy was followed to amplify the full IAPV genome from total RNA extracted from infected bees or viral RNA extracted from particles. The IAPV sequence (GenBank EU224280) was used to design IAPV-BamT7 and IAPV-BamT25 primers (Table S2) with the forward primer including a T7 promoter sequence. Full-length IAPV was cloned into the pJazz vector following the provider's protocol (Lucigen) to produce clone IJJ. An RdRP mutant of the IJJ clone was generated by digestion with XmaI, restriction products were blunt-ended and re-ligated to generate a punctual insertion at coordinate 5,296. This mutant has a C-terminal deletion of the non-structural polyprotein that removes the RdRp coding region. T7 mScript kit (CellScript) was used to produce *in vitro* transcripts from IJJ, the IJJ mutant or from the non-cloned full length IAPV amplified product (IAPV-RTPCR). 5' end capping and 3' end polyadenylation were performed where indicated using the T7 mScript kit (CellScript).

Movies of honey bee cells

Pictures of AmE-711 cells infected with SIDB particles (or non-treated cells) were taken every 15 minutes with a Canon-EOS camera attached to a bright field microscope (10X) for 3-4 days at room temperature. Final movies were

assembled with Windows Movie Maker software (Microsoft Corp.) programmed to 1 sec/frame and final speed of 6X. Conversion to mov files was done in QuickTime Player software (Apple Inc.).

Supplementary REFERENCES

1 Vanengelsdorp, D. *et al.* Colony collapse disorder: a descriptive study. *PLoS ONE* 4, e6481, doi:10.1371/journal.pone.0006481 (2009).

2 Carrillo-Tripp, J. *et al.* Lymantria dispar iflavirus 1 (LdIV1), a new model to study iflaviral persistence in lepidopterans. *J Gen Virol* 95, 2285-2296, doi:10.1099/vir.0.067710-0 (2014).

3 Chen, Y. P., Pettis, J. S., Collins, A. & Feldlaufer, M. F. Prevalence and transmission of honeybee viruses. *Applied and environmental microbiology* 72, 606-611, doi:10.1128/AEM.72.1.606-611.2006 (2006).

4 Desai, S. D., Eu, Y. J., Whyard, S. & Currie, R. W. Reduction in deformed wing virus infection in larval and adult honey bees (Apis mellifera L.) by double-stranded RNA ingestion. *Insect molecular biology* 21, 446-455, doi:10.1111/j.1365-2583.2012.01150.x (2012).

5 Kerr, C. H. *et al.* The 5' untranslated region of a novel infectious molecular clone of the dicistrovirus cricket paralysis virus modulates infection. *J Virol* 89, 5919-5934, doi:10.1128/jvi.00463-15 (2015).

6 Carrillo-Tripp, J., Bonning, B. C. & Miller, W. A. Challenges associated with research on RNA viruses of insects. *Current Opinion in Insect Science* 8, 62-68, doi:http://dx.doi.org/10.1016/j.cois.2014.11.002 (2015).

7 Liu, S., Vijayendran, D., Carrillo-Tripp, J., Miller, W. A. & Bonning, B. C. Analysis of new aphid lethal paralysis virus (ALPV) isolates suggests evolution of two ALPV species. *J Gen Virol* 95, 2809-2819, doi:10.1099/vir.0.069765-0 (2014).
